# Recovering Motor Activation with Chronic Peripheral Nerve Computer Interface

**DOI:** 10.1038/s41598-018-32357-7

**Published:** 2018-09-20

**Authors:** Thomas E. Eggers, Yazan M. Dweiri, Grant A. McCallum, Dominique M. Durand

**Affiliations:** 1 Neural Engineering Center, Biomedical Engineering, Case Western Reserve University, Cleveland, USA; 2 Present Address: Department of Biomedical Engineering, Jordan University of Science and Technology, Irbid, Jordan

## Abstract

Interfaces with the peripheral nerve provide the ability to extract motor activation and restore sensation to amputee patients. The ability to chronically extract motor activations from the peripheral nervous system remains an unsolved problem. In this study, chronic recordings with the Flat Interface Nerve Electrode (FINE) are employed to recover the activation levels of innervated muscles. The FINEs were implanted on the sciatic nerves of canines, and neural recordings were obtained as the animal walked on a treadmill. During these trials, electromyograms (EMG) from the surrounding hamstring muscles were simultaneously recorded and the neural recordings are shown to be free of interference or crosstalk from these muscles. Using a novel Bayesian algorithm, the signals from individual fascicles were recovered and then compared to the corresponding target EMG of the lower limb. High correlation coefficients (0.84 ± 0.07 and 0.61 ± 0.12) between the extracted tibial fascicle/medial gastrocnemius and peroneal fascicle/tibialis anterior muscle were obtained. Analysis calculating the information transfer rate (ITR) from the muscle to the motor predictions yielded approximately 5 and 1 bit per second (bps) for the two sources. This method can predict motor signals from neural recordings and could be used to drive a prosthesis by interfacing with residual nerves.

## Introduction

Advances in neural engineering allow for chronic interfacing with the peripheral nervous system. Selective stimulation of residual nerves in amputees has been shown to restore natural and functional sensation of the phantom limb^1^. An unmet clinical need for amputee patients is the ability to recover motor activation from the nervous system, which would allow for robust and functional restoration of the lost limb.

The only available commercial approach is myoelectric control, in which the electromyograms (EMG) of residual muscles in the arm control the prosthetic limb. EMG can be collected non-invasively and has advanced more rapidly than other modalities. When the amputation is distal to the elbow, functional recovery is high as the muscles that once controlled the wrist and hand remain partially intact. Invasive EMG may provide an even more robust and clinically viable option^2^. However, the functionality of this control method is greatly reduced when the amputation is above the elbow^3^, although advanced machine learning approaches may alleviate this problem^4^. In addition, such devices have no innate way of restoring sensation to the user beyond sensory substitution.

Interfacing directly with the nervous system offers the ability to recover motor activation as well as restore sensation with a single device, as early work in the field showed that residual nerves (and thus cortical areas) retained functional motor and sensory connections years after amputation^5^. Direct cortical interfacing has been explored^6^, but is mostly limited to para-and quadriplegics due to its highly invasive nature. Peripheral nerve interfacing is less invasive and has been studied extensively^7^. Intrafascicular approaches, in which electrodes are implanted within fascicles, offer high selectivity, and have shown promise in both motor recovery and sensory restoration in sub-chronic human trials^8–11^. However, a major drawback from these approaches is the tendency for recording quality to degrade over time. In contrast, extrafascicular approaches such as nerve cuffs have demonstrated long term stability and have been used to study peripheral nerve interfacing in animals^12,13^ and in human trials^14^, most notably for stimulation to restore sensation^1^, but classically record whole nerve activity.

In this study, the Flat Interface Nerve Electrode (FINE), which increases the surface area to volume ratio by gently reshaping the nerve into an ellipse to decrease the distance between the fascicles and an array of electrodes, was used to interface with peripheral nerves. A previously published analysis successfully recorded from two fascicles selectively in the sciatic nerve of a dog^15^. However, only binary signals could be recovered from each fascicle, and classification was performed on large time windows (0.3–0.6 sec) during the portions of the gait when the signals were expected, which would not allow for real time control of a prosthetic limb. To increase the functionality of recovered signals we have utilized significant advances in signal processing that use Bayesian approaches for signal recovery^16–18^. Here we report data showing that a single 16-ch FINE can accurately record nerve signals during normal gait, separate the activity from the main fascicles and selectively predict the activation of two innervated muscles using 100 ms windows, which could more readily translate to a real-time control scheme. We further estimated the information transfer rate of these recovered signals, measuring 5 and 1 bit per second; to the author’s knowledge, these calculations represent the first chronic measurement of information transfer rate for a peripheral nerve. This study demonstrates for the first time that voluntary motor signals correlated with muscle activity can be obtained from a peripheral nerve with a nerve computer interface.

## Results

### Recorded Signals

**Figure 1:**
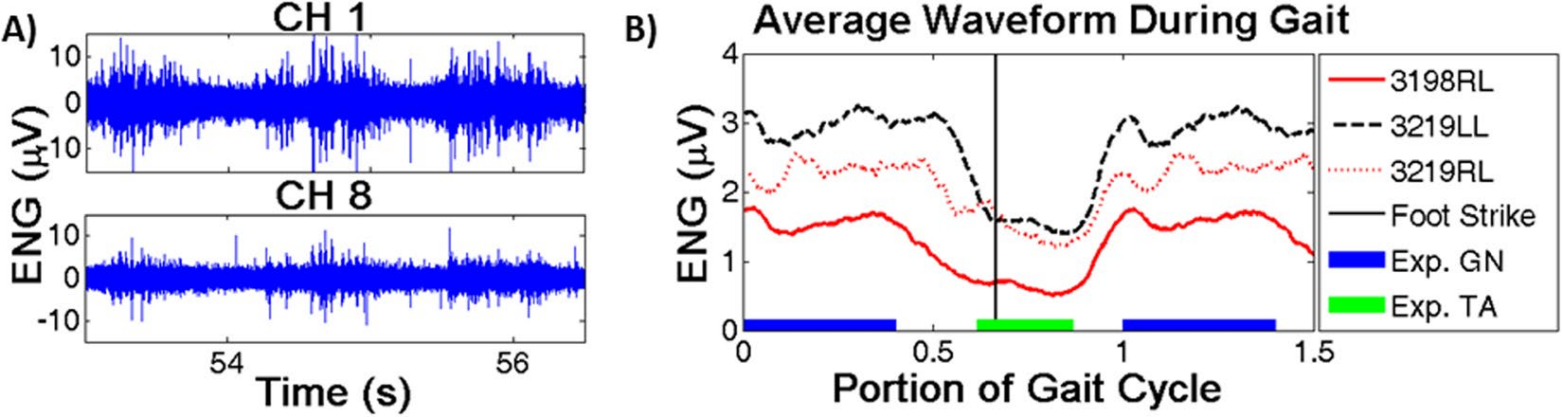
Recorded waveforms. (**A**) Raw recording from two channels on opposite sides of the cuff. Amplitude differences between channels is used to localize activity within the cuff. (**B**) Average waveform envelope for all three study legs (2 weeks post implant). Blue and green bars represent the expected gastroc./tib. ant. activations, while the vertical line represents foot strike. X-axis represents normalized portion of the gait.

Figure 1 shows both some raw data as well as the envelope of the average gait cycle from a single recording session from each of the three legs, 2 weeks post implant. Figure 1A shows approximately 5 s of raw neural activity from one animal. Amplitude differences between channels are used by the algorithm to localize activity for signal extraction. In Fig. 1B, envelopes were created by taking the root-mean-square (RMS) of the signals over 100 ms segments. Gait cycles were normalized to allow direct comparison between animals and show the approximate time the foot lifts off the ground (vertical line) as well as the activation times of the two target muscles, the tibialis anterior (TA) and medial gastrocnemius (GN). The SNR ranged from ~4–7 dB across animals and the impedance of each contact from ~2–4 kΩ, with no downward trend over the implant duration.

### Extracting Motor Activation

**Figure 2:**
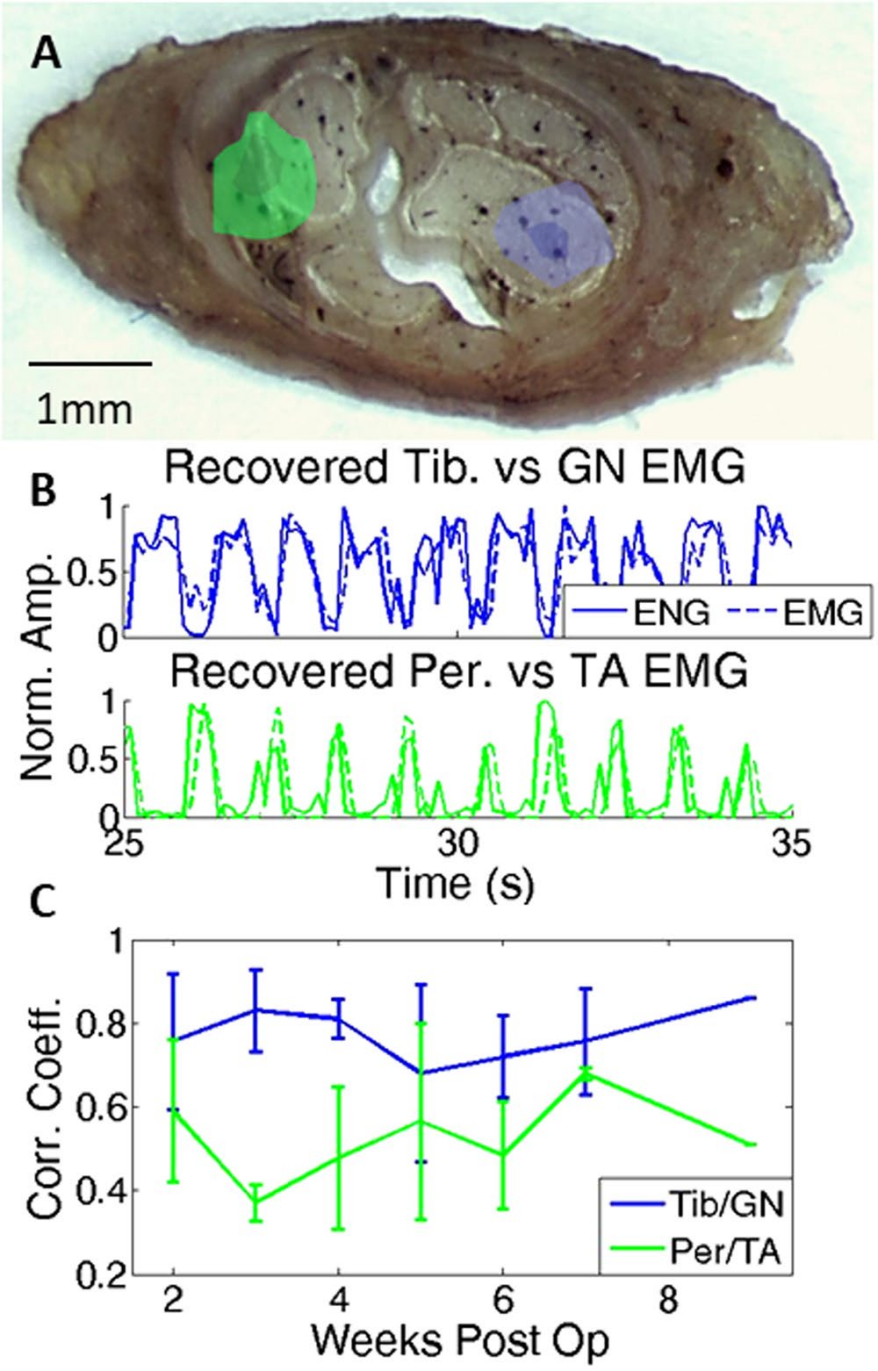
(**A**) Example of nerve with overlaid source localization (3198). Green/blue corresponds to peroneal/tibial nerves. Extra fascicles likely correspond to cutaneous sural nerves. Localization from initial training trial overlaid on histological image after removing cuff. (**B**) Example recording showing recovered neural signals (solid) and corresponding EMG (dash) during 10 s of gait. Data taken two weeks post operation. (**C**) Combined correlation coefficients over two months for three legs (mean ± SD). Week 9 has only 1 data point as the other two lost either EMG (3198) or percutaneous connector (3219RL).

The HBSE algorithm was applied to 16-ch raw ENG for each recording session. The algorithm estimated the source locations as well as the optimal bandwidth and number of pixels for recovery from a training set consisting of 10–20 seconds during locomotion with an additional 5 seconds of baseline. An example of the source localization is overlaid on the nerve cross section (Fig. 2A). The method for overlaying source localization on the nerve cross section has been detailed previously^15^; images are overlaid after removing the cuff and thus represent approximate locations. The optimal pixel sizes and filters varied slightly between animals and trials, typically with a high pass corner frequency of 300 Hz and 9 pixels (~0.2 mm^2^). One leg (3198) required significantly more pixels, ~30–40, to achieve optimal results; the reason for this difference is not known. The algorithm was sensitive to training sets used, likely due to the imprecise method of selecting training samples; thus a full n-fold cross validation was not employed, instead using a single, user-defined segment for training and the remaining segments for testing.

**Figure 3:**
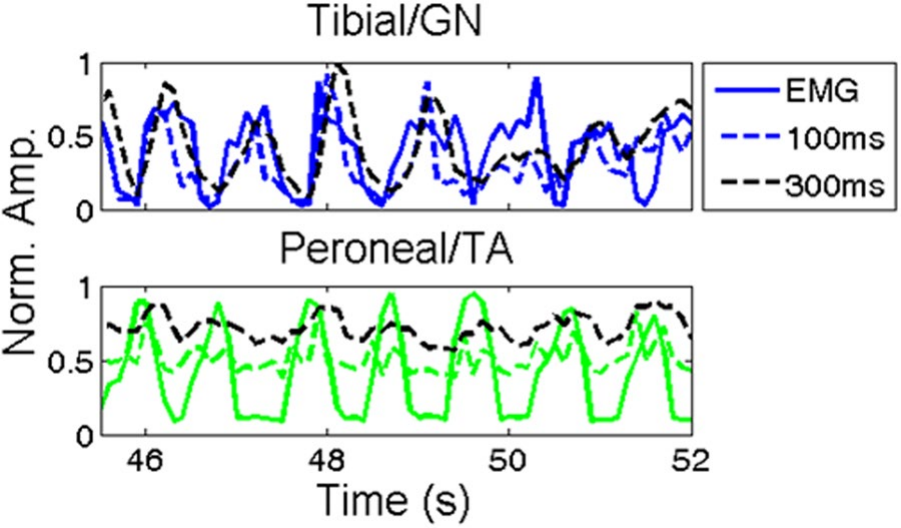
Comparing extracted signals with different windows. The solid lines show zero overlap with 100 ms windows, the black lines show 300 ms processing windows with 200 ms overlap, and the dashed lines show the corresponding EMG. The overlap introduces some delay in the signal, seen more clearly with the tibial/GN, while increasing the reliability of recovery, seen more pronounced in the peroneal/TA signal. The recovered peroneal signal has a lower SNR, which results in an elevated baseline (signals are normalized to 1). Activity recorded 1 month post implant.

**Figure 4:**
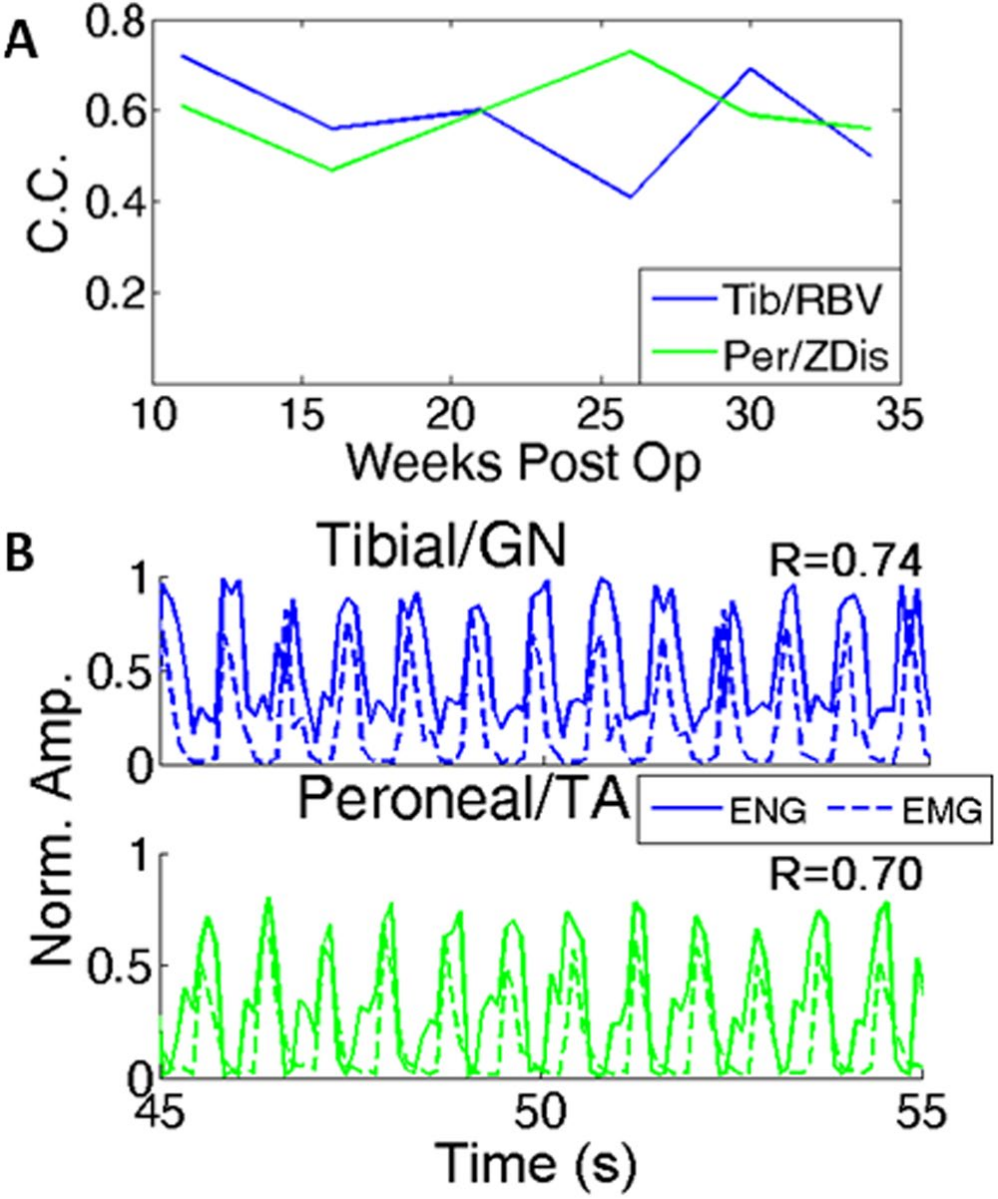
(**A**) Correlation coefficients between extracted neural signals from one animal and the corresponding kinematic variables, ranging from 2 to 7 months post implant. These kinematic variables are the vertical displacement (Zdis) and rectified backward velocity (RBV), and are detailed in Appendix I. (**B**) Example of extracted neural signal (solid) and corresponding EMG (dash) from percutaneous wires 32 weeks post implant.

Figure 2B shows an example of signal recovery from animal 3219LL (left leg) two weeks post implantation (the first recording from this animal). Dashed lines represent the recorded EMG signals while solid lines represent the recovered neural signals, both normalized to 1. This normalization divided the recovered signal by the maximum recovered value and did not adjust the baseline value. The signals return to zero in Fig. 2B, although in other examples (Figs 3 and 4b) the signal does not return to zero as the SNR of these recovered signals is lower. Figure 2C shows the collective data from the three legs under study for the first two months of recordings. The correlation coefficients of all trials between the recovered signals and the corresponding EMG of the GN/TA were 0.76 ± 0.05 and 0.52 ± 0.09, respectively. Over this two month period no downward trend in correlation coefficients are seen (t-test for regression line, p > 0.05).

The processing window length was varied from this initial 100 ms to identify an optimal window length. A scheme which utilizes overlapping windows to increase window sizes has been proposed in myoelectric literature^19^. For this study, the time between EMG outputs was kept constant at 100 ms while the data window of neural activity being analyzed was varied. Figure 3 shows a recording processed with this alternate processing scheme using 300 ms along with the generic scheme for comparison. Table 1 summarizes the results for various time windows, in which each time was compared to the original 100 ms processing window. The highest correlation coefficients were achieved using the 300 ms window, 0.84 ± 0.07 and 0.61 ± 0.12 for the GN/tibial and TA/peroneal signals, respectively.

**Table 1:** Comparing the change in correlation coefficients between different processing time windows.

	20 ms	50 ms	200 ms	300 ms	500 ms
Tibial/GN (%)	−21.2 ± 9.2	−8.7 ± 5.2	5.2 ± 5.9	6.5 ± 9.1	2.1 ± 15.5
Peroneal/TA (%)	−66.0 ± 40.4	−30.8 ± 28.6	23.8 ± 13.6	35.3 ± 18.4	29.4 ± 34.5

The numbers represent the percentage increase/decrease of the correlation coefficient of the ENG/EMG to the 100 ms case (data shown in Fig. 2: 0.76 ± 0.05 and 0.52 ± 0.09 for the tibial/GN and peroneal/TA, repsectively).

### Long-term Correlation with Kinematic Variables

We then sought to determine if some of the kinematic variables involved in the gait motion could be recovered from the neural activity. Optical tracking of the lower limb was obtained and used to estimate muscle function. The vertical position of the foot and the velocity of the limb were extracted from the optical tracking marker data and compared to the EMG from all three legs during trials which simultaneously recorded both signals. These variables represent non-invasive correlates of muscle function (Appendix I).

The long term stability of this signal recovery method was tested on one animal using the above-described kinematic variables as the target signals. Figure 4A shows these correlation measurements over time for 3198, from the period after losing the implanted EMG signals (week 9) until the last viable recording (week 35). During one recording trial 32 weeks post op, bipolar percutaneous hook electrodes were inserted into the target EMG muscles (GN/TA), and is shown in Fig. 4B alongside the extracted neural signals. The recovered signals still matched the recorded EMG, with correlation coefficients of 0.74 and 0.70 for the GN/TA.

### Verification of true neural origin of the activity

**Figure 5:**
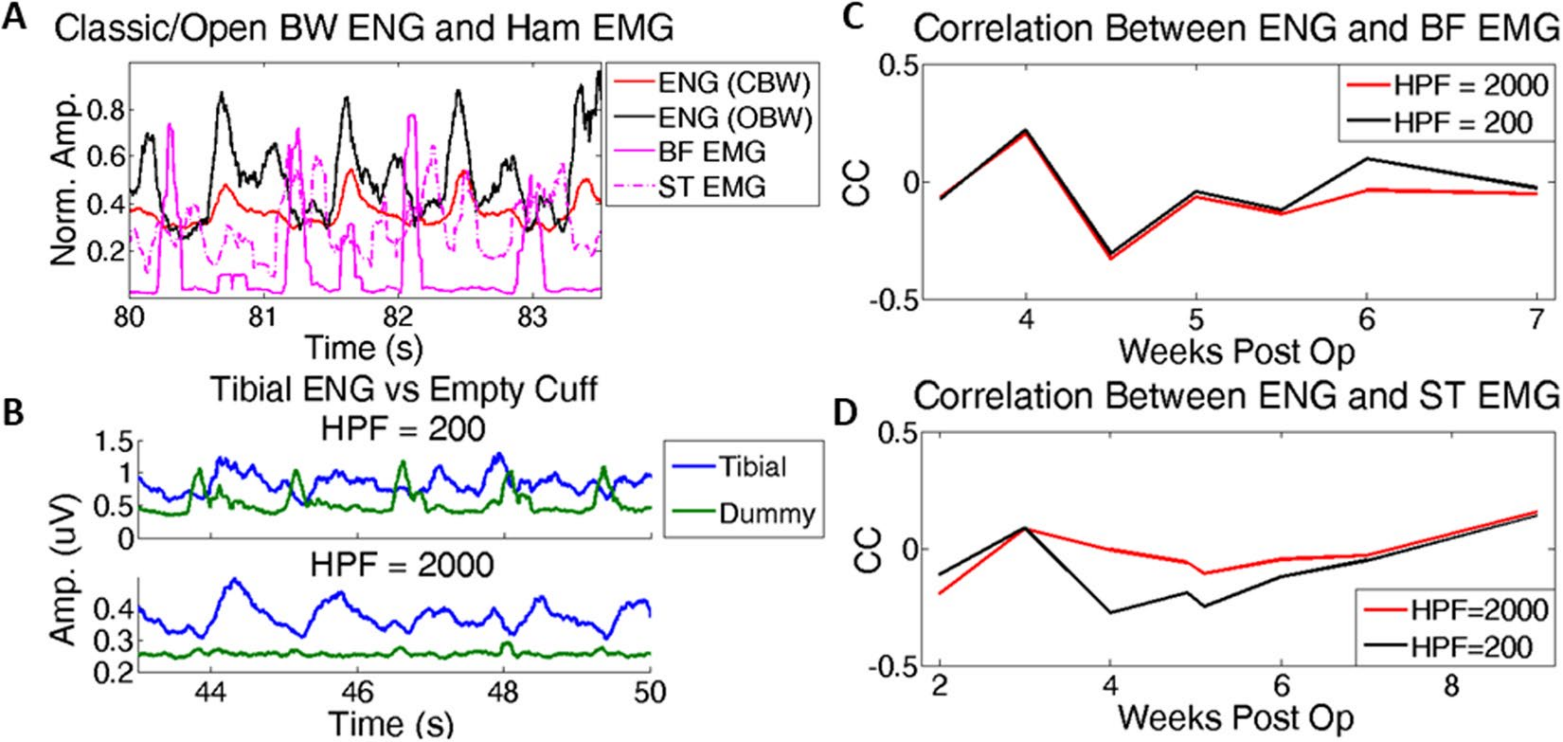
Interfering EMG/ENG shows no correlation. (**A**) Example recording during gait, showing ENG with the classic (HPF = 1 kHz) and open (HPF = 0.2 kHz) bandwidth (BW) as well as the nearby bicep femoris (BF) and semitendinous (ST) EMG. BF EMG is clearly out of phase with both neural plots, while ST EMG shows both in phase and out of phase activity. (**B**) Example of a dummy/empty cuff recording with a tibial nerve recording with two different high pass filters used, 200 and 2000 Hz. The dummy cuff shows a similar out of phase trend as the recorded EMG, and disappears once the lower bandwidth (200–2 kHz) is removed. (**C**,**D**) Correlations between the ENG and recorded EMG (BF/ST, respectively) with the two BWs over the first 2 months of recordings.

The cuff recording electrodes are implanted just above the popliteal fossa and are surrounded by the hamstring muscle. This large muscle is active during gait and could interfere with the ENG recording from the peroneal and tibial fascicles. Therefore we obtained recordings from two hamstring muscles in two different animals and placed an empty (dummy) cuff near the tibial nerve in a third animal. Recordings were obtained simultaneous with neural recordings to investigate the possibility of EMG contamination (see Appendix II for details of methodology). The envelopes of this potentially interfering EMG activity were compared to the average neural waveform to measure any overlap, which would indicate crosstalk. The correlation coefficients between these signals and the neural envelopes were not statistically different from zero (−0.03 ± 0.11 and −0.07 ± 0.15), independent of the bandwidth of the neural signals (Fig. 5). These results indicate that the neural recordings were free of interference from nearby muscles.

### Validating existence of two independent sources

Activation patterns during gait are naturally cyclic, following a predictable pattern. This pattern creates interdependence between these activations, such that one could predict the activity of other muscles with knowledge of a single muscle. To validate the existence of two independent signals in the neural recordings, ICA was conducted. The fast ICA algorithm was run 10 times on the entire recording session for each trial, as large variations in the recovery were seen between trials on the same data; only the best correlation was reported. ICA results were comparable to that of HBSE, with correlations of 0.79 ± 0.07 and 0.43 ± 0.12 for the tibial/GN and peroneal/TA. The HBSE algorithm (0.76 ± 0.05 and 0.52 ± 0.09) performed better than ICA, achieving statistically higher correlations for the TA only (paired t-test, p < 0.05). This analysis confirms the existence of two independent neural signals in the recordings.

### Information Transfer Rate

**Figure 6:**
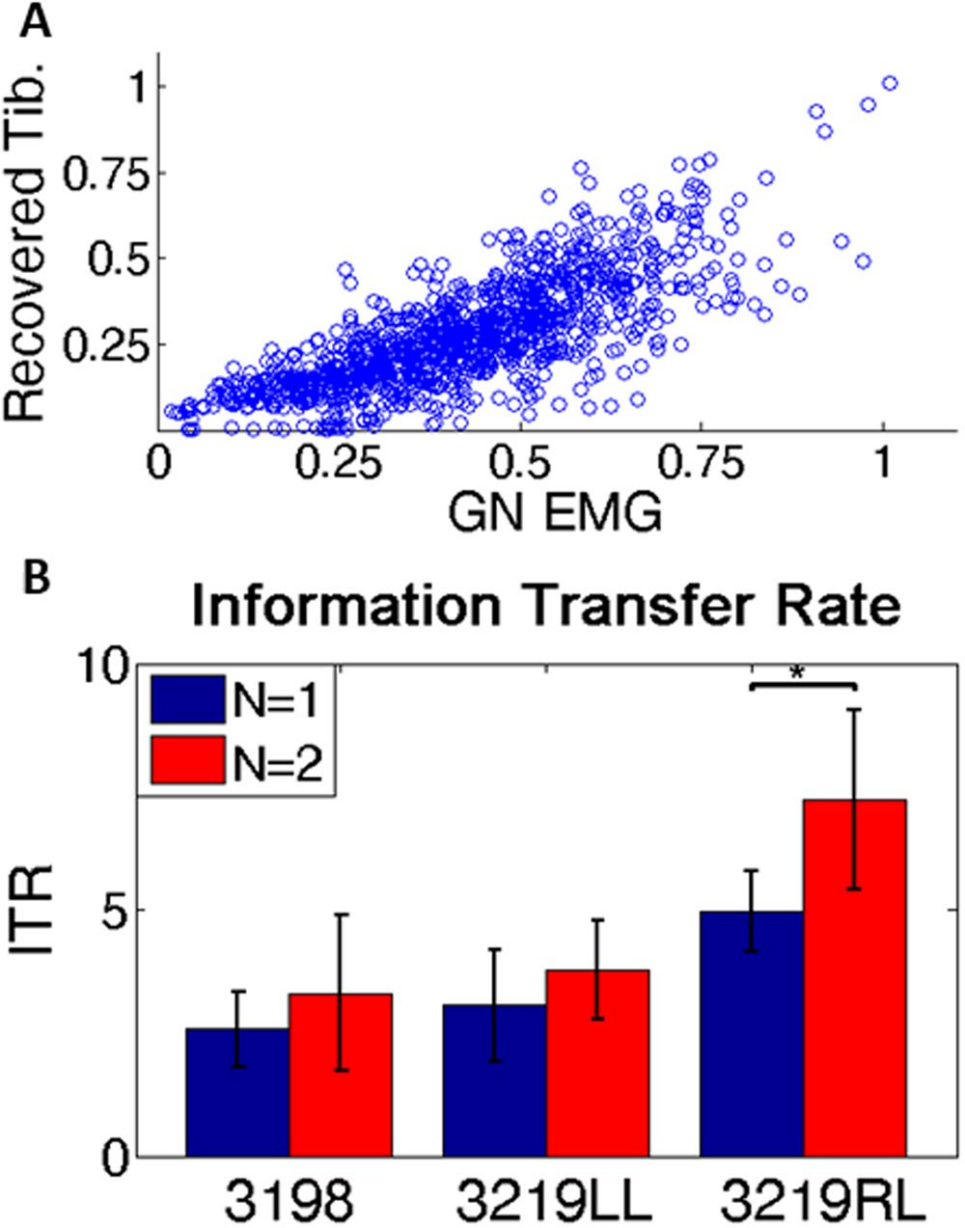
Information calculation. (**A**) Scatter plot of recorded EMG vs recovered signal for one animal (3219LL) for all trials in a two month period. (**B**) Information transfer rate of recovered signal to muscle for all three study legs. The x-axis shows each animal separately with 1 and 2 encoded bits per symbol, and the y-axis the information transfer rate in bits per second. The asterisk represents statistical significance, p < 0.05 (paired t-test).

The correlation coefficient analysis provides a useful metric for signal recovery, although it is not directly comparable to many other prosthetic or BCI control experiments, which tend to measure performance on functional tasks. To create a more universal metric, the amount of recovered information was estimated with the classic ITR calculation (Eq. 1). ITR is commonly employed in BCI tasks, particularly in experiments involving typing^20^, although this metric has also been used in myoelectric studies to measure information about hand reaching movements^21^. Figure 6A shows a scatter plot of the recovered signals and target muscle signals for one animal, demonstrating a linear relationship. Figure 6B shows the ITR for each of the three legs. A paired t-test was performed on each animal for all trials (n = 7). There is a statistically significant difference between the ITR for encoding 1 versus 2 bits (p < 0.05,) only for 3219RL, although an increase was seen in each animal. As the number of bits per symbol increased beyond 2, the ITR rate decreased, approaching 0 at 8 bits. This calculation was also performed for the TA (not shown), yielding approximately 1 bps. This analysis reveals significant information about the muscle activation can be recovered from the nerve.

## Discussion

The results from this study demonstrate for the first time that chronic neural recordings with cuff electrodes can reliably predict motor function and contain significant information. The recordings were obtained in freely moving conditions simulating realistic situations (no faraday cage), albeit restricted to treadmill locomotion due to the percutaneous connector/external amplifiers. Several iterations of the device and amplifiers sequentially improved the recording setup to remove movement artifacts and enable microvolt level recordings in a freely moving animal, see^15^. The novel algorithm, HBSE^18^, allowed the accurate prediction of dynamic motor activation, a significant improvement over the binary recovery demonstrated previously^15^. It is also important to note that the signal recovery paradigm in this study represented continuous motor predictions as opposed to only classifying specified portions of the gait cycle.

### Reproducibility of signals

One advantage of nerve cuff approaches is the homogeneity or predictability of recorded signals, not only over time in the same implant but between subjects (Fig. 1). More selective techniques which recover spiking information record from a subset of the total fiber population. Such selective sampling could potentially lead to variance in functionality, as the recovered spikes are randomly chosen from the fiber population in each individual. Pilot studies recording spike information appear to recover sufficient information to provide some motor control^9,22^, although such interfaces have not yet yielded reliable and predictable recordings required for long-term translation.

### Verification of neural signal recovery

Verifying the neural origin of recorded activity is a critical step in chronic peripheral nerve recordings, particularly with extraneural electrodes as neural spiking information is not available. A common method to quantify external noise involves measuring stimulation artifacts^23–25^. This method is easy to implement in acute preparations but does not directly translate to measuring naturally evoked biological noise in chronic recordings. A superior method involves placing an electrode outside the nerve to record the surrounding biological noise^5^ and comparing to the neural recording. In this study, two steps were taken to verify that the recordings represent the neural signals from two independent sources – EMG interference measurement and ICA. We directly recorded muscle activity from nearby hamstring muscles to demonstrate that little if any of this activity interfered with the neural recordings. An implanted FINE near the nerve (dummy/empty) determined the necessary filter to remove potentially interfering EMG (Fig. 5). A surprising outcome from this analysis revealed that this correlation was independent of filtering bandwidth, allowing us to increase the useable bandwidth and the functional SNR (Appendix II). We attribute this lack of EMG cross-talk to two features of the design: the quasi-tripolar electrode configuration and the presence of thin gold shielding on the outside of the electrode. The ICA analysis shows that the activity from the two fascicles generated independent signals which match the target EMG. The cyclic nature of gait creates dependence between muscles, such that the activation of one muscle can be predicted with knowledge of the other; the unbiased ICA estimate validates that our algorithm is recovering two independent signals. The correlation coefficients shown suggest that improvements with HBSE are small. However, ICA was less reliable at recovering signals and needed the exact EMG signals to determine which components were relevant while HBSE only needed the timing of these signals, making it more translatable to a clinical setting in which an algorithm must be trained on cues.

### Functionality of Signals

A critical question in the study of prosthetic control signals is the determination of the threshold of usability, i.e. answering the question “how good is good enough?” A previous study in which EMG predictions of the upper arm were used to control movement of a temporarily paralyzed limb in primates showed that predictions with correlations between ~0.6–0.9 were sufficient to restore some functional use^26,27^. While these predictions were made from cortical arrays, the same principle still holds and therefore the methods described here from animal trials show the recovered signals can provide independent functional command sources.

To improve the correlation coefficients, the processed window length was varied. However, in order to use these signals to drive a prosthetic hand, constraints on the duration of the window exist. Studies from myoelectric control suggest that 100 ms delay or less is optimal, with longer lags affecting the ability of the user to control the device^19,28^. This constraint led us to first test signal recovery over 100 ms time windows. However, similar to myoelectric control, longer processing windows result in more accurate estimates, creating a tradeoff between accuracy and reaction time. The use of overlapping windows may somewhat address this issue, although longer windows still result in a smoothing of the output. Ultimately a human user would need to test these schemes to determine the optimal configuration.

The quality of the recovered TA/peroneal signal was significantly lower than the GN/tibial signals. We hypothesize that two factors contributed to this lower level of recovery. First, the TA fires in rapid bursts (see Fig. 2B) and the 100 ms analyzing windows may be too long to sample the neural activity accurately. Overlapping windows effectively increases the sampling window, which may explain the pronounced effect of window size on recovery. Second, the TA/peroneal are smaller/contain fewer fibers compared to the GN/tibial and thus produce a smaller signal, making recovery more difficult. As seen in Fig. 1, the recorded signal during dorsiflexion is significantly lower than during the plantarflexion phase, increasing the difficulty of signal recovery.

Another critical component to a viable clinical implant is longevity. Figure 2B demonstrates that signal recovery is stable in all cuffs over the first two months of implantation. Using the proposed kinematic variables, we demonstrate that the recovered signals were stable for the duration of the recording period (7 months) in one animal. A final EMG/neural recording performed at 32 weeks (Fig. 4B) further validates the stability of recovered signals. This long-term stability is not surprising, as similar cuff electrodes have long demonstrated stable stimulation and recording properties, extending out to years of functionality in some studies^1,29^. This study adds further evidence supporting the long-term stability of cuff electrodes.

### Information Content

The information transfer rate represents a more universally quantitative metric about the amount of information extracted from signals. These calculations are common in brain computer interface (BCI) studies where the subject selects letters from a user interface to communicate^20^. We propose a novel information paradigm, in which the brain sends muscle activation levels (the target symbol) to the nerve, with the recorded muscle EMG serving as ground truth. To perform these calculations, several preconditions must be met: the system must be discrete and memoryless, all commands must be equally likely and the accuracy must be the same between symbols, and the errors must be evenly distributed among all symbols. The last precondition is not usually met as the errors are Gaussianly distributed about the target symbol. This violation limits the direct interpretation of the ITR, although these exact conditions frequently are not met in BCI studies^30^. The highest ITR calculated to date with invasive BCI is ~4 bps^20^, comparable to the rate in this study (~4.5). While these numbers fall well short of what the intact human hand is capable of^31^, invasive BCIs have demonstrated the ability to control advanced robotic limbs in laboratory settings^32^, further suggesting that these recovered motor signals are capable of driving a prosthetic limb. The ITR for the TA/peroneal was estimated to be ~1 bps; the precondition of all commands being equally likely is also not met, as the TA is largely inactive with short bursts of activity, further limiting the interpretation of this number. The increase in recovered information encoding more than 1 bit (Fig. 6B) demonstrates that significant information about the activation level of the muscle is recovered, and that dynamic signals beyond the previously analyzed binary signals are recoverable with this nerve computer interface.

### Remaining Challenges

One of the largest hurdles in translating this technology to human use is demonstrating the ability to recover several sources from a nerve which resembles the target nerve (i.e. median, radial and ulnar). The canine sciatic nerve with its small number of fascicles is convenient for testing signal recovery but not representative of human upper limb anatomy, which contain >10 fascicles^33^. Evidence that this technology will scale appropriately, however, is available. First, in silico studies^16^ have shown that 4–5 signals could be recovered from a realistic femoral nerve under the assumption that functionally related fascicles are grouped together. Second, benchtop testing and previous studies has shown that sources can be localized and thus separated within ~500–700 um^16,18^, suggesting that fascicles farther apart than this can be recovered. Thus for a ~3 mm wide cuff/nerve, we can reasonably hope that 3–4 distinct sources can be isolated. Further animal work is necessary to confirm this conjecture.

Another intriguing point is the degree to which the recovered signals follow the dynamic muscle activations. This correlation suggests that the recorded neural signal is largely composed of the motor fibers of the target muscles. However, the sciatic nerve is mixed and innervates not only the target muscles, but several smaller and synergistic muscles of the foot as well as the sensory fibers which actually outnumber the motor signals^34^. One hypothesis as to why the motor signals appear to dominate the recordings relies on the nature of cuff electrodes. Extraneural electrodes record field potentials and thus are more sensitive to synchronous firing of many large fibers (with larger currents^35^), as would occur with motor activity; less synchronous sensory activity from smaller fibers would likely be lost in the underlying noise. However, the presence of large proprioceptive fibers, in particular Ib afferents from Golgi tendon organs, cannot be discounted as contributing to the recorded signal as they would fire concurrent to the motor fibers. The nature of this experimental setup did not allow us to definitively separate these modalities during locomotion.

## Conclusion

In summary, we have demonstrated that multi-channel FINEs are capable of recovering dynamic motor activation from a multi-fascicular nerve. We first demonstrated that the neural recordings are free from interfering EMG from the nearby hamstring muscles and confirmed the existence of two independent signals with ICA analysis. We then showed that stable, reliable prediction of muscle function was possible using these neural signals. The recovered information about the muscle firing demonstrates the viability of using extraneural electrodes as nerve computer interfaces. These signals could be used to drive a prosthetic limb to provide intuitive, proportional control to upper limb amputee patients.

## Methods

### Study Design

Mongrel hounds (n = 2, n = 3 legs) were implanted with FINEs and the study was designed to last 9 months. FINEs were custom-made^36^ and included 16 Pt-Ir recording contacts (0.5 × 1.0 mm) with large references (0.75 × 4.4 mm) on either side for tripolar recordings. Gold sheets were attached and exposed on the outside of the cuff to implement shielding. Briefly, animals walked on a treadmill weekly beginning two weeks after implantation. Neural activity and EMG were recorded during these trials, along with simultaneous optical tracking of the lower limb. Data from three legs in two animals are presented. One animal (3219) destroyed the percutaneous connector two months after implant, and only 9 weeks of data from two legs was collected. The other animal (3198) destroyed its percutaneous connector after 7 months of implantation.

### Surgical Implantation

All surgical procedures were approved by the Case Western Reserve University’s IACUC, and all experiments were performed in accordance with the IACUC guidelines. Animals were initially anesthetized with Propofol, intubated and maintained with isoflurane (2–4%). Heating pads maintained body temperature while heart rate, CO_2_ and blood pressure were continuously monitored. Initial incisions were made along the midline of the back distal to the shoulder blades to place two percutaneous, titanium ports. An incision was then made on the dorsal side of both hind limbs above the hamstrings, and FINEs were tunneled to these sites. Sciatic nerves were exposed by separating the biceps femoris and semitendinosus muscles, and then 2–3 cm of the nerves proximal to the bifurcation of the tibial/peroneal branches were separated from surrounding tissue to place the cuffs. Cuffs were closed with silk sutures and lead wires were tacked down to nearby muscle tissue before closing the incisions. Intramuscular recording EMG electrodes were then implanted in the medial gastrocnemius (GN) and tibialis anterior (TA) of all three legs. These electrodes were bipolar with a barb on the end to fix them in the tissue. Additionally, one animal had an electrode implanted in the biceps femoris (BF, 3198) and one in the semitendinous (ST, 3219 LL). In the third leg two additional single channel FINEs were implanted, one on the tibial nerve and the other closed and left beside the tibial nerve with nothing inside (herein called the dummy cuff). All incision sites were monitored daily for 2 weeks until sutures were removed.

### Recording sessions

Animals were placed on a manually-powered treadmill and walked at a moderate, consistent pace (roughly 1–2 mph) during recordings. These recording sessions occurred once per week and typically consisted of 1–2 minute trials of recording, with 1–2 trials per session. A custom-made preamplifier board utilizing super-β instrumentation amplifiers (AD8421, Analog Devices, Inc.) directly connected to the custom percutaneous port, which recorded quasi-tripolar neural signals and bipolar EMG signals using the titanium port as ground. An AlphaLab recording system (AlphaOmega, Inc) further amplified and digitized the signals, sampling at 22.3 kHz. Prior to recording, optical tracking markers were placed above and below the hock (ankle) and the 3D positions were monitored simultaneous with the recordings (OptiTrack, Natural Point). Subsequent processing was performed offline using Matlab (R2012, MathWorks).

### Signal Processing

Neural activity was processed with the Hybrid Bayesian Signal Extraction (HBSE)^37^ to extract two sources of activity. This algorithm works over predefined window segments, initially 100 ms, and first estimates neural activity using beamforming. Beamforming creates a transformation matrix which maps recorded neural activity to the 2D transverse plane through the nerve at the level of the contacts; this plane consisted of 10 × 35 pixels and was created with finite element modeling^38^. An iterative Bayesian algorithm then refines this estimate by utilizing knowledge of baseline and interfering sources from training. Recovered signals represent the average estimated signal from an area associated with each fascicle, determined from a training set.

To train this algorithm, the number of sources must be defined by the user, and periods of activity reflecting solely the desired source must be provided. To identify times of expected plantarflexion and dorsiflexion, thresholding of the EMG of the GN and TA was employed to isolate periods of single source activity. From here, several more free parameters of the algorithm were set, namely the location and number of pixels to average for each source and the bandwidth (BW) to be used. Both of these parameters were chosen to maximize the signal recovery on the training data and then measured on the validation set. To measure the performance of signal recovery, the Pearson correlation coefficient (R) between the recorded signal and the corresponding EMG (GN/tibial and TA/peroneal) was calculated over the entire validation set, resulting in one coefficient per trial/week. For weeks with 2 trials in the same animal, validation was performed on segments from both recordings and reported as one correlation coefficient. The reported numbers for all data represent mean ± standard deviation.

Independent component analysis (ICA) was also applied to these recordings. A fast ICA algorithm was downloaded and applied to the testing sets (Fast ICAv2.5, 2005). Data was whitened before applying ICA using the singular value decomposition (SVD) method. The number of components was left as the number of channels, and those that best matched the EMG were selected (no training/testing).

### Information Transfer Rate

The information transfer rate (ITR) between the recovered signals and the recorded EMG was calculated for each trial. Preliminary results of this calculation can be found in^37^. To perform this calculation, signals were encoded into bits by normalizing all signals from 0 to 1 and dividing them into several segments, corresponding to a number of bits (i.e. four segments corresponding to 2 bits). The ITR was then calculated with Eq. 1:

1 $$ITR=R\ast ({\mathrm{log}}_{2}N+P\ast {\mathrm{log}}_{2}P+(1-P)\,{\mathrm{log}}_{2}[(1-P)/(N-1)])$$

where R is the rate of classification (10 Hz), N is the number of states encoded by each symbol and P is the accuracy. P is calculated as the percent of encoded recovered signals which match the encoded muscle signal.

## Electronic supplementary material

Supplemental Information

## Data Availability

The datasets generated during and/or analyzed during the current study are available from the corresponding author on reasonable request.
