# Supplementary material for: Recovering Motor Activation with Chronic Peripheral Nerve Computer Interface
Source: Sci Rep. 2018 Sep 20;8:14149. doi: 10.1038/s41598-018-32357-7 (PMC6148292; doi:10.1038/s41598-018-32357-7)
Supplement: Supplementary file 1 — Supplemental Information [file 41598_2018_32357_MOESM1_ESM.docx]

Supplemental Material

Title:

Recovering Motor Activation with Chronic Peripheral Nerve Computer Interface

Authors:

Thomas E. Eggers^1^, Yazan M. Dweiri^2^, Grant A. McCallum^1^, Dominique M. Durand^1*^

Appendix I

*Kinematic Variables for Predicting EMG Activations*

Optical tracking data was recorded with the OptiTrack System (Natural Point). Six markers, three on the lower ankle and three above the ankle joint, were tracked in a 3D coordinate system at 120Hz. To create the GN surrogate signal, the x-value of all six markers were first averaged together to create the variable x_pos. The change in x at each step was then calculated using the Matlab function diff(), and the resulting variable was inverted and half-wave rectified. Finally, this signal was shifted back in time by 100ms to correspond in time with the GN. This variable is referred to in the text as the rectified backward velocity (RBV). The second variable corresponding to the TA was created by first averaging the z-value of the three markers on the foot below the ankle, z_pos. This signal was smoothed using a 1s averaging window, z_smooth, and then soft thresholding was applied to remove the DC drift from z_pos resulting in the final signal referred to as z position (ZPos) in the text.

Fig. S1A shows an example trial with these two variables, Z displacement (ZDis) and rectified backward velocity (RBV), plotted next to their corresponding muscle (TA/GN). This same measurement was taken in all three study animals, with 5-7 trials at least one week apart for each animal. Data is lumped, as no changes over time are expected for this correlation. Each source had a relatively high correlation with low variance, 0.75±0.1 and 0.80±0.05 for the GN/TA, respectively, and is plotted in Fig. S1B.


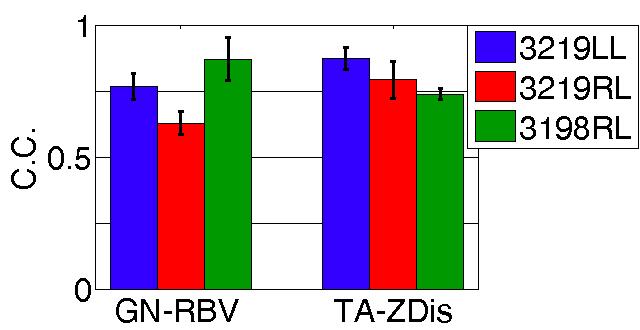

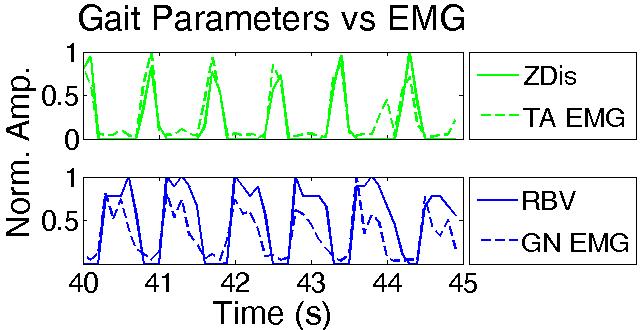


Fig. S1: Kinematic variables and EMG. A) Example of extracted kinematic variables against their corresponding EMG for treadmill walking. B) Lumped correlation coefficients between kinematic variables and EMG for three animals. Each bar represents 5-7 trials.

**A**

**B**

Appendix II - Interfering EMG

*Correlation results between interfering EMG and ENG*

To investigate the possibility of EMG contamination, recordings from two hamstring muscles in two different animals and a dummy cuff (see below) in a third were taken simultaneous to neural recordings. Potential EMG interference would come from the muscles directly surrounding the cuff, the biceps femoris (BF) and/or semitendinous (ST) muscles, and would correlate in time with the neural recording (the semimembranous [SM] is also near the sciatic nerve, although the bifurcation level in the animals implanted occurred between only the BF and ST). An example recording of the neural activity and EMG of these two muscles is shown in Fig. 5A. EMG for this trial was recorded with percutaneous hook electrodes. Neural activity is shown in two bandwidths, one high-pass filtered above 1kHz (‘classic BW’) and again with a high-pass filter above 200Hz (‘open BW’). The neural signals were acquired from 100-9000Hz, and additional filtering was performed with a zero-phase digital filter. The EMGs are largely out of phase with the neural signals, with near zero correlation coefficients. In one animal, a ‘dummy’ cuff was implanted, which was a single channel FINE not implanted on a nerve but closed and placed near the implant site. Another single channel FINE was implanted on the tibial nerve alongside the dummy cuff. Fig. 5B shows the open BW for these two signals. The dummy cuff recorded some activity, although out of phase with the tibial signal. Post-processing revealed that a 2kHz (not 1kHz) high-pass filter was necessary to remove all signal from the dummy cuff. Subsequent recordings (not shown) show a similar trend. To determine potential interference in the 16 channel FINEs, the correlation coefficients between the raw neural and BF/ST EMG were calculated for two bandwidths, with a high-pass filter at 200Hz and 2kHz. Two kilohertz was chosen over the classical BW as it was previously shown to remove potential EMG from the raw cuff recordings. These correlation coefficients for the two bandwidths are shown in Fig. 5C and D. These traces represent two different animals (3198 and 3219LL), as only a single hamstring EMG was recorded in each of these two animals due to limitations in the number of percutaneous connections. Neither correlation was statistically greater than 0, nor was there any difference between the two (paired t-test, p>0.05).

*Effect of Opening the Bandwidth*

The experimental setup employed here allowed the EMG interference from nearby muscles to be investigated. The initial aim was to determine the optimal bandwidth which rejected interfering EMG while maximizing the neural signal. Filtering the lower bandwidth to remove residual EMG has long been employed in cuff recordings, although no systematic study of muscle interference has been conducted in a chronic preparation leading to varied bandwidths employed in cuff recordings, ranging from 0.7-3kHz to 1.5-10kHz in chronic recordings ^11,33^; we called the 1kHz high pass the ‘classical’ bandwidth in this study as it was roughly the average cutoff frequency of previous works. Testing this aim required that the hamstring EMG and recorded neural activity occur independently or out of phase with each other. Fortunately, these signals weren’t naturally correlated within the gait cycle, allowing us to compare the two during normal locomotion. Using the single channel dummy cuff results (Fig. 5B), we concluded that a high pass filter of 2kHz was necessary to truly eliminate potential EMG interference. Our results show no correlations using either this stringent 2kHz or the ‘open’ bandwidth using 200Hz (Fig. 5C/D), implying the interference was not present in either case. This conclusion allowed the filter settings to become a tunable parameter as opposed to a fixed constraint. The initial design aimed only to show that minimal interference existed after filtering, although this analysis revealed the unexpected result that the interference appears to be rejected independent of filtering.

This EMG rejection was accomplished by a combination of the tripolar setup and external shielding. In the ideal world the tripolar design alone eliminate any interference ^34^, although the precise conditions for perfect rejection are never met in practice ^35^. To further reduce interference an external shielding scheme was utilized ^19^. In simulation as well as benchtop testing, this shielding has been shown to reduce EMG interference by ~80% ^31^. It is important to note that rejection also likely depends on the fit of the cuff around the nerve and the degree of closure, with ill fitting (i.e. too large) or poorly closed cuffs being potentially more susceptible to interference. In this study the cuffs were designed to fit closely to the nerve, with the cuff perimeter to nerve circumference ratio from approximately 1.1-1.5. To close the cuffs, simply suturing the flaps together appears sufficient. Together these practices appear to effectively eliminate any EMG interference from the recorded signal.

*Increasing SNR with Open Bandwidth*

To characterize the effect of increasing the BW on the recorded signal’s SNR, the SNR of the raw neural signals was calculated for each recording for both BWs for all three legs in the first two months, and is shown in Fig. S2. A small although significant (paired t-test, p<0.05) increase of 2.9 dB in SNR is observed for the open vs classic BW. SNR was measured as the ratio of the average stance phase RMS to a baseline (i.e. standing) RMS.


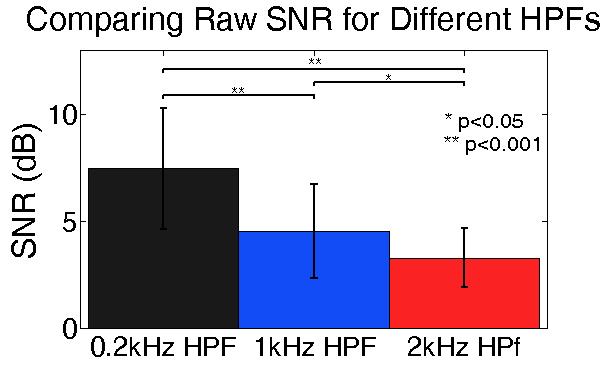


Fig. S2: SNR of neural signals over the first two months in three animals (SNR calculated over the same datasets). The low pass filter is set at 8kHz for all three scenarios. Increasing the bandwidth below 2kHz increases the raw recorded SNR for both the 1-8kHz and 0.2-8kHz bandwidths.
